# The three‐year impact of the Affordable Care Act on disparities in insurance coverage

**DOI:** 10.1111/1475-6773.13077

**Published:** 2018-10-30

**Authors:** Charles Courtemanche, James Marton, Benjamin Ukert, Aaron Yelowitz, Daniela Zapata, Ishtiaque Fazlul

**Affiliations:** ^1^ Department of Economics Gatton College of Business and Economics University of Kentucky Lexington Kentucky; ^2^ National Bureau of Economic Research Cambridge Massachusetts; ^3^ Institute of Labor Economics (IZA) Bonn Germany; ^4^ Department of Economics Andrew Young School of Policy Studies Georgia State University Atlanta Georgia; ^5^ Healthcore Inc. Wilmington Delaware; ^6^ Leonard Davis Institute of Health Economics Philadelphia Pennsylvania; ^7^ Impaq International Washington District of Columbia

**Keywords:** gender/sex differences in health and health care, health care financing/insurance/premiums, health policy/politics/law/regulation, medicaid, racial/ethnic differences in health and health care, state health policies

## Abstract

**Objective:**

To estimate the impact of the major components of the ACA (Medicaid expansion, subsidized Marketplace plans, and insurance market reforms) on disparities in insurance coverage after three years.

**Data Source:**

The 2011‐2016 waves of the American Community Survey (ACS), with the sample restricted to nonelderly adults.

**Design:**

We estimate a difference‐in‐difference‐in‐differences model to separately identify the effects of the nationwide and Medicaid expansion portions of the ACA using the methodology developed in the recent ACA literature. The differences come from time, state Medicaid expansion status, and local area pre‐ACA uninsured rates. In order to focus on access disparities, we stratify our sample separately by income, race/ethnicity, marital status, age, gender, and geography.

**Principal Findings:**

After three years, the fully implemented ACA eliminated 43% of the coverage gap across income groups, with the Medicaid expansion accounting for this entire reduction. The ACA also reduced coverage disparities across racial groups by 23%, across marital status by 46%, and across age‐groups by 36%, with these changes being partly attributable to both the Medicaid expansion and nationwide components of the law.

**Conclusions:**

The fully implemented ACA has been successful in reducing coverage disparities across multiple groups.

## 1 INTRODUCTION

Prior to the implementation of the primary components of the Affordable Care Act (ACA) in 2014, there were well‐documented disparities in insurance coverage along multiple dimensions, such as age, race, income, family structure, and geography.^1^, ^2^ The primary components of the ACA, including the individual mandate, subsidized Marketplace coverage, and state Medicaid expansions, were designed to reduce health insurance coverage disparities by moving the U.S. closer to universal coverage.^3^ The purpose of this paper was to examine the extent to which the ACA reduced disparities in coverage after three years (2014‐2016).

While gains in insurance coverage after the ACA have been well documented, few papers in this literature examine how the ACA affected coverage disparities. One recent paper estimates the first‐year impact of the ACA on coverage using difference‐in‐difference‐in‐differences (DDD) models where the differences come from time, state Medicaid expansion decisions, and pre‐ACA local area uninsured rates.^4^ This strategy leverages the propensity for universal coverage initiatives to provide the most intense “treatment” in local areas with the highest prereform uninsured rates.^5^, ^6^ Using data from the American Community Survey (ACS), the authors find that the ACA increased coverage by an average of 5.9 percentage points in Medicaid expansion states compared to 2.8 percentage points in nonexpansion states in 2014.^4^ In subsample analyses, they show that the fully implemented ACA (including the Medicaid expansion) reduced the coverage disparity between college graduates and those with a high school diploma or less by 11.4%, and that between whites and nonwhites by 14%.^4^ The paper also finds greater gains in coverage for young adults and unmarried individuals, which had lower pre‐ACA coverage rates than older adults and married individuals, respectively.^4^

Another recent paper uses the same research strategy and data from the Behavioral Risk Factor Surveillance System (BRFSS), finding that the ACA reduced the coverage disparity between those with incomes above versus below the median by 38%.^7^ A third paper uses ACS data through 2015 and leverages variation in state Medicaid expansion decisions, pre‐ACA eligibility requirements, and subsidy rates across the income distribution.^8^ They find that coverage gains from the Medicaid expansion and premium subsidies are larger among childless adult couples than among single adults or adults with children, but the increase from the individual mandate is largest among singles.^8^

Other studies focus only on the ACA's Medicaid expansion, using simpler difference‐in‐differences (DD) models to compare changes in insurance coverage over time between Medicaid expansion and nonexpansion states. One paper includes 2015 data from the ACS and shows that the Medicaid expansion reduced the coverage disparity between 19‐ to 26‐ and 56‐ to 64‐year‐olds by 15%, while the disparity between Hispanics and non‐Hispanic whites only fell by 4%.^9^ Another paper, also using data through 2015, finds that the Medicaid expansion led to smaller gains among low‐income Hispanics than other low‐income individuals, implying a widened disparity.^10^ Other papers provide evidence that the Medicaid expansion increased insurance coverage among those with low incomes or levels of education, implying reduced disparities relative to individuals with higher socioeconomic status.^11^, ^12^ One study's focus is on the impact of the ACA in a single state, Kentucky, finding that much of the reduction in the state's uninsured rate is due to large coverage gains from areas with higher concentrations of poverty.^13^

We contribute to this literature by using the DDD method described above and elsewhere^4^, ^7^, ^14^ to uncover the causal impact of the 2014 ACA provisions, both with and without the Medicaid expansion, on coverage disparities after three years. Changes in coverage disparities are evaluated by stratifying our sample by income, race/ethnicity, marital status, age, gender, and geography. Data come from the American Community Survey (ACS) between 2011 and 2016. The ACS includes multiple categories of insurance coverage, allowing us to evaluate how the ACA affected coverage disparities via changes to both private and public coverage. In addition, the ACS is a large enough survey to precisely estimate the effects for states and many local areas, given that it includes approximately 3 000 000 observations per year and relatively narrow geographic identifiers. Finally, the mandatory nature of the ACS reduces concerns about sample selection among respondents.

Our primary hypothesis is that, in its first three years, the ACA significantly reduced insurance coverage disparities across demographic groups. We contribute to the literature on the ACA's coverage effects in multiple ways. First, we are, to our knowledge, the first to quantify the impacts of the ACA on disparities using three years of post‐ACA implementation data (2014‐2016). One recent study examines the effect on the uninsured rate after three years using the BRFSS, but does not specifically examine disparities.^14^ Second, in contrast to the BRFSS, the ACS allows us to examine how changes in sources of coverage, such as employer‐sponsored and individually purchased private coverage and Medicaid, drove any changes in disparities. Third, in contrast to other recent work,^9^, ^10^ our approach allows us to estimate the impact of the fully implemented ACA, rather than just focusing on the Medicaid expansion. Fourth, we examine disparities along a new dimension: residence in rural vs. urban locations.

## 2 DATA

The ACS is a nationally representative survey administered by the Census Bureau sampling approximately 1% of the U.S. population annually. Participation is mandatory, and the survey can be completed online or through the mail. In terms of geography, the ACS identifies all 50 states and the District of Columbia, and additionally identifies localities known as Public Use Microdata Areas (PUMAs). PUMAs are approximately 2300 areas of at least 100 000 people nested entirely within a state. Our primary sample consists of 19‐ to 64‐year‐olds from calendar years 2011 to 2016, which results in over 3 000 000 individuals per year. By starting our sample period in 2011, we aim, as in other recent work,^4^ to measure only the effects of the package of ACA provisions taking effect in 2014, as opposed to also capturing the effect of the 2010 dependent coverage mandate that required insurers to allow dependents to remain on their parents’ insurance plans until the plan year following their 26th birthday. This mandate has already been studied extensively in prior research.^15^, ^16^, ^17^, ^18^, ^19^

We create several binary outcome variables based on the ACS insurance coverage questions: any insurance, any private insurance (either employer sponsored or directly purchased), employer‐sponsored insurance, directly purchased insurance, Medicaid, and any other coverage. We define other coverage as neither private nor Medicaid coverage. These categories are not mutually exclusive due to the possibility of multiple sources of coverage.

In order to exploit within‐state variation in pre‐ACA uninsured rates in 2013 to identify the impact of the national components of the ACA, we would ideally simply use the PUMA classification system included in the ACS. Unfortunately, the PUMA definitions changed during our sample period due to new boundaries introduced in the 2010 Census. To address this problem, we follow a recent paper^4^ and use both the old and new PUMA classification systems to identify core‐based statistical areas (CBSAs), which we then use as our local areas. If a CBSA spans multiple states, we define a different local area for the parts of the CBSA in each state. In addition, we create additional local areas for the non‐CBSA portion of each state, in order to prevent respondents who do not live in a CBSA from being dropped from the sample. We classify non‐CBSA local areas as “rural” and CBSA local areas as “urban.” Our dataset consists of 630 local areas that each contain between 356 and 78 781 ACS respondents in 2013, with a median of 1020 and a mean of 2811 respondents. This implies that our pre‐ACA uninsured rates are computed from a reasonably large sample in each local area.

By 2016, a total of 32 states expanded their Medicaid program via the ACA.^20^ The majority of these states, 25 in all, expanded Medicaid in January 2014. Michigan (April) and New Hampshire (August) expanded later in 2014. Pennsylvania (January), Indiana (February), and Alaska (September) expanded Medicaid during 2015. Finally, Montana (January) and Louisiana (July) expanded Medicaid during 2016. We assign the starting date of these states’ Medicaid expansions in our expansion indicator accordingly.

Our “demographic” controls include dummies for age (25‐29, 30‐34, 35‐39, 40‐44, 45‐49, 50‐54, 55‐59, and 60‐64, with 19‐24 being the omitted base category), female, race/ethnicity (non‐Hispanic black, Hispanic, and other race/ethnicity, with non‐Hispanic white being the omitted category), foreign‐born, and U.S. citizenship status. Our “family structure” controls include dummies for married and the number of children 18 and under in the household (one, two, three, four, and five or more, with zero being the omitted category). Our “economic” controls consist of dummies for education (high school degree, some college, and college graduate, with less than a high school degree as the omitted category), household income (dummies for each 10‐point increment of income as a percentage of the Federal Poverty Level (FPL), with the highest category including everyone over 500%), whether the respondent reports her primary occupation as student, and whether the respondent is unemployed, as well as one continuous variable: the Bureau of Labor Statistics’ annual state unemployment rate. Finally, we include interactions of the post‐treatment dummy with indicators of whether states set up their own private insurance exchanges (as opposed to using the federal exchange) and whether these exchanges experienced glitches.^20^, ^21^ These variables serve as proxies for harder‐to‐measure aspects of state involvement with the ACA, such as the degree of state outreach.

Table 1 provides pretreatment means and standard deviations of the dependent variables of interest measuring insurance coverage, while Table S1 does the same for the controls. We also stratify into four groups based on whether the respondent's state expanded Medicaid and whether her local area's pretreatment uninsured rate was above or below the median for individuals in the sample. Table 1 shows that 79% of the sample was covered by some type of insurance in the baseline year of 2013, including 11% with Medicaid and 60% with employer‐provided coverage. For both the high‐ and low‐uninsured rate subgroups, individuals in Medicaid expansion states were slightly more likely to be covered by some type of insurance in 2013 than those in nonexpansion states, with the differences being driven entirely by Medicaid coverage. Our DDD model will account for such baseline differences.

**Table 1:** Descriptive statistics for insurance coverage

	Full sample	Medicaid expansion; at or above median baseline uninsured	Medicaid expansion; below median baseline uninsured	Nonexpansion; at or above median baseline uninsured	Nonexpansion; below median baseline uninsured
Any insurance coverage	0.792 (0.406)	0.748 (0.434)	0.847 (0.359)	0.729 (0.444)	0.837 (0.370)
Any private	0.668 (0.471)	0.616 (0.486)	0.719 (0.450)	0.610 (0.488)	0.722 (0.448)
Employer‐sponsored	0.598 (0.490)	0.544 (0.498)	0.650 (0.477)	0.543 (0.498)	0.649 (0.477)
Individually purchased	0.094 (0.292)	0.093 (0.291)	0.094 (0.292)	0.091 (0.287)	0.100 (0.299)
Medicaid	0.106 (0.307)	0.115 (0.319)	0.121 (0.326)	0.090 (0.286)	0.090 (0.286)
Other	0.032 (0.176)	0.030 (0.169)	0.024 (0.152)	0.041 (0.198)	0.038 (0.191)

*Note:*

Standard deviations are in parentheses.

**Figure 1:**
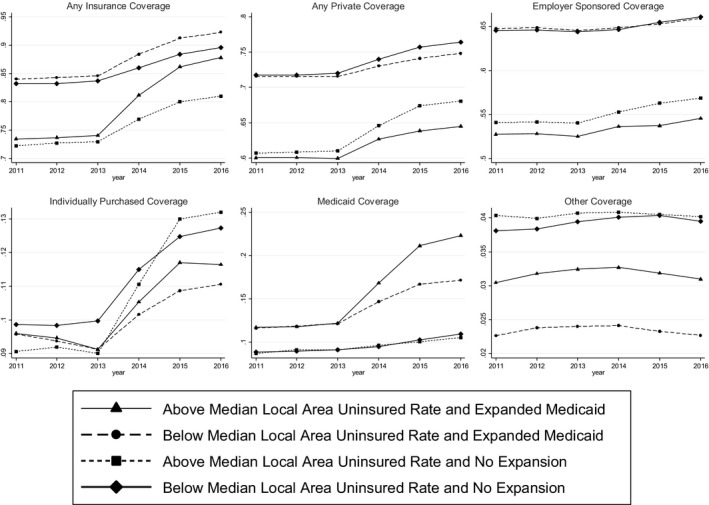
Changes in insurance coverage over time

Figure 1 presents changes in our coverage measures between 2011 and 2016, stratified into the same four groups. With six insurance outcomes and four groups per outcome, there are a total of 24 lines. In general, the pre‐ACA trends do not appear to differ meaningfully by state Medicaid expansion status or local area pre‐ACA uninsured rate. This provides preliminary support for the use of the pre‐ACA uninsured rate and Medicaid expansion variables as sources of identification in our DDD model. Figure 1 shows that the probabilities of having any coverage, privately purchased coverage, any private coverage, and Medicaid increased in 2014 and continued to grow over the following two years.

## 3 METHODS

In order to uncover the causal impact of the ACA on coverage disparities after three years, we follow the recent ACA literature by estimating DDD models with the differences coming from time, state Medicaid expansion decisions, and pre‐ACA local area uninsured rates.^4^, ^7^, ^14^ Our baseline DDD regression equation is given by equation (1) below. In order to examine coverage disparities, we estimate this model separately for different subsamples, such as separately for males and females.

(1) $$\begin{array}{cc} y_{\mathrm{iast}}= & \gamma_{0}+\gamma_{1}\left({\mathrm{UNINSURED}}_{\mathrm{as}}\times {\mathrm{POST}}_{t}\right)+\gamma_{2}\left({\mathrm{MEDICAID}}_{S}\times {\mathrm{POST}}_{t}\right)+\\ & \gamma_{3}\left({\mathrm{UNINSURED}}_{\mathrm{as}}\times {\mathrm{MEDICAID}}_{S}\times {\mathrm{POST}}_{t}\right)+\gamma_{4}X_{\mathrm{iast}}+\theta_{t}+\alpha_{\mathrm{as}}+\varepsilon_{\mathrm{iast}} \end{array}$$

where $y_{\mathrm{iast}}$ is an indicator of insurance coverage for individual *i* in local area *a* in state *s* in year *t*,* POST*_*t*_ is an indicator for whether period *t* is in the postreform period of 2014 or later, ***X***_***iast***_ is a vector of control variables previously described, *MEDICAID*_*s*_ is an indicator for whether state *s* participated in the ACA's Medicaid expansion, *UNINSURED*_*as*_ is the 2013 (pre‐ACA) uninsured rate in local area *a* within state *s*, $\theta_{t}$ denotes year fixed effects, $\alpha_{\mathrm{as}}$ denotes local area fixed effects, and $\varepsilon_{\mathrm{iast}}$ is a standard error term.

The term *POST*_*t*_ is not separately included in Equation (1) since it is absorbed by the time fixed effects, while the terms *UNINSURED*_*as*_ **× *MEDICAID*_*st*_ are not separately included since they are absorbed by the local area fixed effects.

The effect of the ACA without the Medicaid expansion is given by *γ*_1_ × *UNINSURED*_*as*_, which means it is assumed to be zero in a (hypothetical) area with a 0% uninsured rate at baseline and to increase linearly as the pre‐ACA uninsured rate rises.^4^ Similarly, the effect of the Medicaid expansion alone is given by $\gamma_{3}\times UNINSURED_{\mathrm{as}}\times MEDICAID_{\mathrm{st}}$, meaning it is zero in nonexpansion states (where $MEDICAID_{\mathrm{st}}=0$) and $\gamma_{3}\times UNINSURED_{\mathrm{as}}$ in expansion states (where $MEDICAID_{\mathrm{st}}=1$). We consider $\gamma_{2}$ to represent unobserved confounders rather than capturing part of the expansion's causal effect, since the Medicaid expansion should not causally affect coverage in an area with a 0% baseline uninsured rate. The effect of the “fully implemented” ACA, that is, in Medicaid expansion states, combines the impacts of the Medicaid and non‐Medicaid components: $\gamma_{1}\times UNINSURED_{\mathrm{as}}+\gamma_{3}\times UNINSURED_{\mathrm{as}}$. In our results, we report the predicted effect of the ACA at the sample mean pretreatment uninsured rate. Formally, this predicted effect is given by $\gamma_{1}\times \bar{UNINSURED_{\mathrm{as}}}$ in nonexpansion states and $\gamma_{1}\times \bar{UNINSURED_{\mathrm{as}}}+\gamma_{3}\times \bar{UNINSURED_{\mathrm{as}}}$ in expansion states. For each subsample of interest, we recompute the pretreatment uninsured rate using only individuals within that particular subsample.^4^

## 4 RESULTS

Tables 2, 3, 4 report the implied effects of the ACA at the average pre‐ACA uninsured rate based on coefficient estimates from the DDD regression described by equation (1) for each coverage outcome. The top panel of Table 2 gives the implied effects that come from our full nonelderly adult sample, while the subsequent panels stratify the sample in different ways. The first row in each panel shows the pre‐ACA uninsured rate, which we use to calculate the pre‐ACA disparities in coverage. Indicators of statistical significance are given at the 0.1%, 1%, and 5% levels. For each regression, we separately report: (a) the implied effects of the Medicaid expansion alone, and (b) the fully implemented ACA, which includes the Medicaid expansion as well as the individual mandate, subsidized Marketplace coverage, etc. This allows for easier comparison to previous work that focused on the Medicaid expansion and not the national components of the ACA.

**Table 2:** Implied effects of the ACA at mean pretreatment uninsured rate for full sample and income and race subsamples

	Any insurance	Any private	Employer‐sponsored	Individually purchased	Medicaid	Other
*Panel I: Full sample*						
Nonelderly adults aged 19‐64 (pretreatment uninsured rate = 0.203, sample size = 10 537 667)						
Medicaid expansion	0.050*** (0.011)	−0.009 (0.009)	0.003 (0.007)	−0.010 (0.012)	0.062*** (0.008)	0.001 (0.001)
Full ACA (w/Medicaid)	0.087*** (0.005)	0.028*** (0.006)	0.017*** (0.004)	0.012*** (0.003)	0.062*** (0.007)	0.001 (0.001)
*Panel II: Income subsamples*						
Under 138% FPL (Pretreatment Uninsured Rate = 0.395, sample size = 1 949 375)						
Medicaid expansion	0.170*** (0.033)	−0.031 (0.018)	−0.007 (0.016)	−0.024 (0.019)	0.216*** (0.036)	−0.003 (0.003)
Full ACA (w/Medicaid)	0.169*** (0.029)	0.018 (0.011)	0.015 (0.009)	0.002 (0.007)	0.154*** (0.031)	0.002 (0.002)
138%‐400% FPL (Pretreatment Uninsured Rate = 0.237, sample size = 4 137 149)						
Medicaid expansion	0.056*** (0.016)	0.011 (0.015)	0.023 (0.016)	−0.008 (0.016)	0.047** (0.015)	0.001 (0.002)
Full ACA (w/Medicaid)	0.105*** (0.013)	0.066*** (0.014)	0.050*** (0.011)	0.019*** (0.006)	0.042** (0.013)	−0.001 (0.001)
Over 400% FPL (Pretreatment Uninsured Rate = 0.067, sample size = 4 482 022)						
Medicaid expansion	0.012** (0.004)	0.002 (0.006)	0.006 (0.009)	−0.002 (0.007)	0.010* (0.004)	−0.001 (0.002)
Full ACA (w/Medicaid)	0.028*** (0.003)	0.019*** (0.005)	0.008 (0.005)	0.010*** (0.003)	0.010* (0.004)	0.001 (0.002)
*Panel III: Race/Ethnicity subsamples*						
Non‐Hispanic white (Pretreatment Uninsured Rate = 0.144, sample size = 7 149 482)						
Medicaid expansion	0.051*** (0.006)	−0.002 (0.006)	0.004 (0.008)	−0.004 (0.007)	0.058*** (0.008)	−0.002 (0.001)
Full ACA (w/Medicaid)	0.077*** (0.005)	0.019*** (0.005)	0.010** (0.004)	0.007* (0.003)	0.067*** (0.007)	−0.003*** (0.001)
Non‐white (Pretreatment Uninsured Rate = 0.306, sample size = 3 388 185)						
Medicaid expansion	0.070*** (0.020)	−0.014 (0.019)	0.005 (0.009)	−0.015 (0.020)	0.083*** (0.013)	0.006* (0.002)
Full ACA (w/Medicaid)	0.115*** (0.009)	0.039*** (0.009)	0.025** (0.008)	0.018** (0.006)	0.075*** (0.012)	0.004* (0.002)

*Notes:*

Results are effects of the ACA on the proportion of residents with the specified type of insurance, evaluated at the mean pretreatment uninsured rate. Standard errors, heteroskedasticity‐robust and clustered by state, are in parentheses. *** indicates statistical significance at 0.1% level, ** at 1% level, and * at 5% level. Sampling weights are used. All regressions include area and time fixed effects and the full set of controls.

**Table 3:** Implied effects of the ACA at mean pretreatment uninsured rate for marital status and age subsamples

	Any insurance	Any private	Employer‐sponsored	Individually purchased	Medicaid	Other
*Panel I: Marital status subsamples*						
Married (pretreatment uninsured rate = 0.141, sample size = 5 978 285)						
Medicaid expansion	0.032*** (0.008)	−0.005 (0.006)	0.0010 (0.004)	−0.006 (0.008)	0.038*** (0.005)	0.001 (0.001)
Full ACA (w/Medicaid)	0.060*** (0.003)	0.022*** (0.003)	0.010*** (0.003)	0.012*** (0.002)	0.040*** (0.003)	0.001 (0.001)
Unmarried (pretreatment uninsured rate = 0.272, sample size = 4 559 382)						
Medicaid expansion	0.080*** (0.013)	−0.010 (0.013)	0.005 (0.011)	−0.012 (0.015)	0.093*** (0.013)	0.001 (0.002)
Full ACA (w/Medicaid)	0.120*** (0.009)	0.035*** (0.009)	0.025*** (0.006)	0.011* (0.004)	0.089*** (0.011)	0.001 (0.001)
Panel II: Age subsamples						
Ages 19‐26 (pretreatment uninsured rate = 0.270, sample size = 1 562 121)						
Medicaid expansion	0.077*** (0.015)	−0.008 (0.014)	0.006 (0.011)	−0.011 (0.012)	0.090*** (0.013)	0.001 (0.002)
Full ACA (w/Medicaid)	0.124*** (0.009)	0.031** (0.009)	0.024** (0.008)	0.009* (0.004)	0.097*** (0.012)	0.003* (0.001)
Ages 27‐34 (pretreatment uninsured rate = 0.256, sample size = 1 667 573)						
Medicaid expansion	0.054*** (0.012)	−0.012 (0.011)	0.006 (0.008)	−0.004 (0.009)	0.070*** (0.013)	0.001 (0.003)
Full ACA (w/Medicaid)	0.093*** (0.009)	0.020* (0.008)	0.010 (0.006)	0.010* (0.004)	0.076*** (0.009)	0.002 (0.003)
Ages 35‐49 (pretreatment uninsured rate = 0.201, sample size = 3 330 941)						
Medicaid expansion	0.035*** (0.010)	−0.012 (0.009)	−0.002 (0.006)	−0.008 (0.010)	0.046*** (0.008)	0.002 (0.002)
Full ACA (w/Medicaid)	0.073*** (0.005)	0.026*** (0.005)	0.017*** (0.004)	0.010*** (0.003)	0.048*** (0.006)	0.001 (0.001)
Ages 50‐64 (pretreatment uninsured rate = 0.145, sample size = 3 977 032)						
Medicaid expansion	0.036*** (0.007)	−0.005 (0.005)	0.008 (0.008)	−0.010 (0.012)	0.044*** (0.005)	−0.002 (0.002)
Full ACA (w/Medicaid)	0.070*** (0.004)	0.030*** (0.004)	0.015*** (0.003)	0.016*** (0.003)	0.044*** (0.003)	−0.002 (0.002)

*Notes:*

Results are effects of the ACA on the proportion of residents with the specified type of insurance, evaluated at the mean pretreatment uninsured rate. Standard errors, heteroskedasticity‐robust and clustered by state, are in parentheses. *** indicates statistical significance at 0.1% level, ** at 1% level, and * at 5% level. Sampling weights are used. All regressions include area and time fixed effects and the full set of controls.

**Table 4:** Implied effects of the ACA at mean pretreatment uninsured rate for gender and rural/urban subsamples

	Any insurance	Any private	Employer‐sponsored	Individually purchased	Medicaid	Other
*Panel I: Gender subsamples*						
Women (pretreatment uninsured rate = 0.186, sample size = 5 473 836)						
Medicaid expansion	0.064*** (0.011)	−0.006 (0.009)	0.007 (0.010)	−0.011 (0.010)	0.071*** (0.013)	0.002 (0.001)
Full ACA (w/Medicaid)	0.096*** (0.008)	0.030*** (0.007)	0.023*** (0.006)	0.008* (0.004)	0.067*** (0.011)	0.002 (0.001)
Men (pretreatment uninsured rate = 0.223, sample size = 5 094 710)						
Medicaid expansion	0.057*** (0.011)	−0.003 (0.010)	0.010 (0.011)	−0.008 (0.015)	0.067*** (0.012)	−0.003 (0.002)
Full ACA (w/Medicaid)	0.097*** (0.008)	0.040*** (0.010)	0.028*** (0.005)	0.017*** (0.015)	0.063*** (0.010)	−0.001 (0.002)
*Panel II: Rural vs urban subsamples*						
Rural (pretreatment uninsured rate = 0.212, sample size = 1 964 610)						
Medicaid expansion	0.081*** (0.016)	−0.015 (0.013)	−0.012 (0.008)	−0.004 (0.012)	0.098*** (0.020)	0.001 (0.004)
Full ACA (w/Medicaid)	0.120*** (0.014)	0.018 (0.011)	0.014** (0.005)	−0.004 (0.010)	0.110*** (0.017)	−0.004 (0.003)
Urban (pretreatment uninsured rate = 0.203, sample size = 8 603 936)						
Medicaid expansion	0.056*** (0.011)	−0.004 (0.009)	0.011 (0.011)	−0.010 (0.013)	0.064*** (0.011)	−0.001 (0.002)
Full ACA (w/Medicaid)	0.091*** (0.008)	0.036*** (0.008)	0.026*** (0.006)	0.013*** (0.004)	0.058*** (0.010)	0.001 (0.001)

*Notes:*

Results are effects of the ACA on the proportion of residents with the specified type of insurance, evaluated at the mean pretreatment uninsured rate. Standard errors, heteroskedasticity‐robust and clustered by state, are in parentheses. *** indicates statistical significance at 0.1% level, ** at 1% level, and * at 5% level. Sampling weights are used. All regressions include area and time fixed effects and the full set of controls.

The first column of the top panel of Table 2 suggests that at the average pre‐ACA uninsured rate, the Medicaid expansion increased the proportion of residents with insurance coverage by 5 percentage points over the three‐year period of 2014‐2016. In comparison, the fully implemented ACA led to an 8.7 percentage point increase in coverage, implying that the package of nationwide reforms contributed the remaining 3.7 percentage points. The remaining columns examine sources of coverage, where we consider any source of private coverage, any employer‐sponsored (ESI) plan, any individually purchased plan, Medicaid, and any other coverage source. Our results suggest that in an area with the mean pre‐ACA uninsured rate, the fully implemented ACA is predicted to increase private coverage by 2.8 percentage points. This is driven by increases in both ESI (1.7 percentage points) and individually purchased coverage (1.2 percentage points). The full ACA increased Medicaid coverage by 6.2%, all of which comes from the Medicaid expansion component of the law. The effects of the Medicaid expansion on having any private coverage, ESI, and individually purchased insurance reported are all statistically insignificant.

The other two panels of Table 2 stratify the sample into subsamples separately by income and race. Since each subsample must contain enough 2013 respondents to accurately compute uninsured rates at the local area level, we are constrained to a maximum of two or three subsamples per stratification in order to obtain meaningfully precise estimates. For income, we consider three groups: those with income under 138% of the FPL, those between 138% and 400%, and those above 400%. The lowest income group was made eligible for Medicaid in states that expanded their Medicaid programs via the ACA. Additionally, those between 100% and 138% of the FPL were eligible for subsidized Marketplace coverage with sliding scale premiums in states that did not expand Medicaid. The middle‐income group was made eligible for subsidized Marketplace coverage with sliding scale premiums in all states. The highest income group was also able to purchase Marketplace coverage in all states, but was not eligible for a subsidy. In 2013, the uninsured rate for those in the highest income group was 6.7%, while it was 39.5% for the lowest income group.

According to the first column of Table 2, this 32.8 percentage point coverage gap was reduced by 15.8 percentage points (= 17 percentage point reduction for the lowest income group—1.2 percentage point reduction for the highest income group) by the Medicaid expansion. This represents a 48% reduction in the low‐income coverage gap. The fully implemented ACA, which includes the Medicaid expansion, but also influences the coverage of higher income individuals through the national components of the ACA, reduced the low‐income coverage gap by 43%.

Turning to sources of coverage in the remaining columns, several results emerge. First, the gain in coverage among low‐income individuals occurred completely via Medicaid coverage in Medicaid expansion states. Moreover, some of the coverage expansion among middle‐ and high‐income individuals occurred via Medicaid coverage even though these income ranges were not eligible for the expansion.

We next examine the race stratification in the third panel of Table 2. The racial coverage gap in 2013 was 16.2 percentage points, with nonwhites having an uninsured rate of 30.6%, as compared to 14.4% for non‐Hispanic whites. Our results suggest that the Medicaid expansion reduced the 16.2 percentage point coverage gap by 1.9 percentage points (12%), while the fully implemented ACA reduced the gap by 3.8 percentage points (23%). The results for source of coverage show that the larger gains for nonwhites occur across the board, as the effects of the full ACA on all types of coverage are larger for them than for whites.

The top panel of Table 3 examines disparities of coverage by marital status. In 2013, unmarried individuals had a 27.2% uninsured rate, while married individuals had a 14.1% uninsured rate. This unmarried coverage gap was reduced by 4.8 percentage points (37 %) by the Medicaid expansion, while the fully implemented ACA reduced this coverage gap by 6 percentage points (46%). The shrinking gap is attributable to larger gains in employer‐provided and Medicaid coverage among unmarried individuals.

The bottom panel of Table 3 splits the sample into four age‐groups: 19‐26 years of age, 27‐34 years of age, 35‐49 years of age, and 50‐64 years of age. The rationale for separating 19‐ to 26‐year‐olds from 27‐ to 34‐year‐olds is that the former was previously affected by the earliest major ACA coverage expansion to take effect, the 2010 dependent coverage mandate. Depending on the mandate's effectiveness, it is possible that the effect of the 2014 ACA provisions that we study could be weaker among 19‐ to 26‐ than 27‐ to 34‐year‐olds. That said, the results show that those aged 19‐26 years still had the highest uninsured rate (27.0%) among the age‐groups in 2013, three years after the dependent coverage mandate took effect. Those aged 50‐64 had the lowest uninsured rate of 14.5%, for a young adult coverage gap of 12.5 percentage points. The Medicaid expansion reduced this coverage gap by 4.1 percentage points (33%), and all 2014 ACA provisions together reduced it by 5.4 percentage points (43%). The larger gain among 19‐ to 26‐year‐olds is driven by a much larger increase in Medicaid coverage (9.7 percentage points for the full ACA compared to 4.4 percentage points among 50‐ to 64‐year‐olds).

The top panel of Table 4 stratifies sample by gender. We did not observe as large an initial coverage gap by gender (22.3% uninsured rate for males vs. 18.6% for females). The results suggest that the Medicaid expansion actually increased the size of this coverage gap by 19% since it reduced the uninsured rate for females by a greater degree than it did for males. Conversely, the fully implemented ACA reduced the size of the gender coverage gap by 3%.

The bottom panel of Table 4 stratifies our sample by urban vs. rural location. Rural individuals are generally considered a vulnerable population when it comes to health care access. The uninsured rate in 2013 was 21.2% for rural nonelderly adults as compared to 20.3% for urban nonelderly adults, so the initial disparity in terms of insurance coverage was about 1 percentage point. The fully implemented ACA reduced this disparity by 2.9 percentage points, 2.5 percentage points of which comes from the Medicaid expansion.

Tables 2, 3, 4 only report impacts of the ACA at the relevant mean pretreatment uninsured rate, which is 20.3% for the full sample and varies for each subsample of interest. This approach masks considerable heterogeneity in the law's effects since local area pretreatment uninsured rates varied widely, ranging from 3% to 53% with a standard deviation of 7% for the full sample. Figure S1 therefore shows how the predicted changes in coverage vary across this range of uninsured rates in both expansion states (as indicated by “Full ACA”) and nonexpansion states (as indicated by “ACA Without Medicaid Expansion”). The top left graph shows that the predicted impact of the full ACA on the probability of having any coverage reached as high as 22.6 percentage points at the highest sample pretreatment uninsured rate (53%). In contrast, without the Medicaid expansion the maximum effect was only 9.5 percentage points. The “Medicaid coverage” graph predicts increases in Medicaid coverage that reach as high as 16.1 and 0.1 percentage points in expansion and nonexpansion states, respectively.

Similarly, Tables S2‐S4 display the impact of the Medicaid expansion and the fully implemented ACA for the mean of the lower and higher portion of the uninsured rates for all subsamples. These results suggest that for practically every type of coverage, the larger the proportion of the subsample uninsured in 2013, the larger the gain in coverage. For example, among individuals with income below 138% of the FPL, the mean pretreatment uninsured rate for the upper half of their uninsured rate distribution is 46.3% and the mean for the lower half of the distribution is 33%. Table S2 reports that the fully implemented ACA is predicted to increase coverage by 20 percentage points at the mean of the upper half of the uninsured rate distribution of this group, while predicted to increase coverage by 14 percentage points at the mean of the lower half of the distribution.

## 5 DISCUSSION

The primary components of the ACA were designed to reduce insurance coverage disparities by moving the U.S. closer to universal coverage. This paper is the first to use three years of post‐ACA implementation data (2014‐2016) to estimate the impact of the law on disparities across different categories of coverage along several dimensions, including income, race, marital status, age, gender, and residence in rural vs. urban locations. We find that the ACA reduced the coverage disparity by income by 43% and that this was entirely driven by the Medicaid expansion that was specifically targeted at low‐income childless adults. Conversely, we find that both the Medicaid expansion and nationwide provisions of the ACA contributed to the 23%, 46%, and 36% reductions in coverage disparities by race, marital status, and age, respectively. The ACA did not meaningfully influence the coverage gap by gender, while it closed and actually reversed the small rural coverage gap. Ultimately, we conclude that the ACA substantially reduced coverage disparities along several important dimensions, but that sizeable disparities still remain across income, race, marital status, and age‐groups.

The fact that the ACA's impacts on disparities cannot be fully attributed to the Medicaid expansion along most dimensions illustrates the importance of implementing econometric techniques that capture other aspects of the law. The DDD models we report on this paper are causally interpretable based on the identifying assumptions that, conditional on the controls, if the ACA had not occurred: (a) changes in coverage in the postreform period would not have been correlated with prereform uninsured rates, and (b) any differential changes in coverage in the postreform period between expansion and nonexpansion states would not have been correlated with prereform uninsured rates.

One limitation of our work is that we cannot test these identifying assumptions directly. However, we can test them indirectly with an event study model that interacts the treatment variables with the full set of year fixed effects. This allows us to trace out the effects of the treatment variables over time, relative to a base year of 2013. We would expect around 5% of the coefficient estimates of interest in the pretreatment years 2011 and 2012 to be statistically significant at the 5% level merely by chance. Substantially more significant findings for these “placebo tests” could indicate a problem with the identifying assumption. In Tables S5‐S7, we present the results from the event study models for the full sample and all subsamples. Across the three tables, the interactions of the treatment variables with 2011 and 2012 are significant 14% of the time (55 out of 384), which is somewhat greater than the expected 5%. However, for many subsamples the model performs quite well. Nearly half of the placebo test failures (23 out of the 55) are concentrated among the coefficients on $UNINSURED_{\mathrm{as}}\times POST_{t}$ for the age subsamples. Our main results for the effects of the non‐Medicaid expansion components of the ACA among the age subsamples should therefore be interpreted with the most caution.

Another limitation is that our disparity analyses assume that the subsamples are exogenously determined. Income is one source of stratification that might seem particularly likely to adjust endogenously in response to the 2014 ACA provisions. Two recent papers found little impact of the ACA Medicaid expansions on work effort, implying that the effect on income should be minimal.^11^, ^22^ Another found that while labor market outcomes in the aggregate were not significantly affected by the ACA, labor force participation reductions in areas with higher potential exchange enrollment were offset by increases in labor force participation in areas with higher potential Medicaid enrollment.^23^ In order to examine whether our particular ACA treatment variables influence income, the first column of Table S8 presents the results of our baseline regression model estimated with household income (measured relative to the poverty line) as the dependent variable. The results suggest that there was no statistically significant effect of the Medicaid expansion or the fully implemented ACA on individual income.

Similarly, it is also possible that the 2014 ACA provisions had some impact on marital decisions. A recent doctoral dissertation found that the Massachusetts coverage expansion of 2006—which featured a combination of insurance market regulations, individual and employer mandates, subsidies, and health insurance exchanges that served as the model for the ACA—had only small effects on marriage and divorce rates.^24^ In Table S8, we estimate our baseline regression model separately with indicators of being married, of being newly married during the past 12 months, of being newly divorced during the past 12 months, and of being newly married or divorced in the past 12 months as dependent variables. The results suggest that there was no statistically significant effect of our ACA treatment variables on these outcomes. In addition, we replicate our main analyses after dropping individuals from the sample that had any change in their marital status in the last 12 months. The results, reported in Table S9, are very similar to the results presented previously. While the available evidence therefore suggests that our assumption of exogenous stratification is plausible, it is of course not possible to establish this definitively.

With those limitations in mind, our results are broadly consistent with those reported in the Medicaid expansion literature^9^ in that both the Medicaid expansion and the fully implemented ACA generally reduce but do not eliminate coverage disparities. These results imply that full repeal of the ACA would exacerbate these disparities. Additionally, it is possible that changes to the ACA after 2016, including regulatory changes, such as Medicaid work requirements, and the elimination of the individual mandate, would lead to further changes in disparities. For example, our finding that the Medicaid expansion eliminated 43% of the coverage gap across income groups is likely to change if Medicaid work requirements, that would be expected to potentially reduce enrollment,^25^ are widely implemented. Thus, more work is needed to examine the impact of the ACA as economic conditions change and the ACA itself changes.
