# Supplementary material for: The three‐year impact of the Affordable Care Act on disparities in insurance coverage
Source: Health Serv Res. 2018 Oct 30;54(Suppl 1):307–16. doi: 10.1111/1475-6773.13077 (PMC6341207; doi:10.1111/1475-6773.13077)
Supplement: Supplementary file 2 [file HESR-54-307-s002.docx]

**Figure A1. ACA Effect on Insurance Coverage at Pre-Treatment Uninsured Rate**
