# Supplementary material for: The three‐year impact of the Affordable Care Act on disparities in insurance coverage
Source: Health Serv Res. 2018 Oct 30;54(Suppl 1):307–16. doi: 10.1111/1475-6773.13077 (PMC6341207; doi:10.1111/1475-6773.13077)
Supplement: Supplementary file 3 [file HESR-54-307-s003.docx]

**Table A1. Pre-Treatment Summary Statistics for Control Variables**

|  | Full Sample | Medicaid Expansion; at or above Median Baseline Uninsured | Medicaid  Expansion; below Median Baseline Uninsured | Non- Expansion; at or above Median Baseline Uninsured | Non-  Expansion; below Median Baseline Uninsured |
| --- | --- | --- | --- | --- | --- |
| *Demographic controls* |  |  |  |  |  |
| Age dummies (19-24 is omitted base category)^a^ | | | | | |
| Age 25-29 | 0.110  (0.313) | 0.114  (0.318) | 0.110  (0.312) | 0.109  (0.311) | 0.110  (0.313) |
| Age 30-34 | 0.109  (0.311) | 0.111  (0.315) | 0.108  (0.310) | 0.108  (0.311) | 0.108  (0.310) |
| Age 35-39 | 0.104  (0.305) | 0.106  (0.308) | 0.102  (0.302) | 0.106  (0.308) | 0.101  (0.302) |
| Age 40-44 | 0.111  (0.314) | 0.111  (0.314) | 0.111  (0.314) | 0.112  (0.316) | 0.109  (0.311) |
| Age 45-49 | 0.114  (0.318) | 0.111  (0.314) | 0.116  (0.320) | 0.113  (0.317) | 0.114  (0.317) |
| Age 50-54 | 0.118  (0.323) | 0.114  (0.317) | 0.122  (0.327) | 0.116  (0.321) | 0.121  (0.326) |
| Age 55-59 | 0.109  (0.311) | 0.104  (0.305) | 0.111  (0.314) | 0.107  (0.310) | 0.112  (0.315) |
| Age 60-64 | 0.095  (0.294) | 0.092  (0.289) | 0.097  (0.295) | 0.096  (0.294) | 0.096  (0.294) |
| Female | 0.510  (0.500) | 0.505  (0.500) | 0.509  (0.500) | 0.513  (0.500) | 0.510  (0.500) |
| Race/ethnicity dummies (non-Hispanic white is omitted base category) | | | | | |
| Non-Hispanic black | 0.121  (0.326) | 0.069  (0.253) | 0.107  (0.309) | 0.171  (0.377) | 0.123  (0.328) |
| Hispanic | 0.163  (0.369) | 0.298  (0.458) | 0.115  (0.319) | 0.187  (0.390) | 0.066  (0.247) |
| Other | 0.080  (0.271) | 0.106  (0.308) | 0.101  (0.302) | 0.052  (0.222) | 0.052  (0.222) |
| Foreign born | 0.171  (0.376) | 0.251  (0.434) | 0.180  (0.384) | 0.153  (0.360) | 0.088  (0.283) |
| US citizen | 0.904  (0.295) | 0.855  (0.352) | 0.907  (0.290) | 0.905  (0.293) | 0.949  (0.220) |
| Disability | 0.102 (0.303) | 0.099  (0.298) | 0.093  (0.290) | 0.116  (0.320) | 0.104  (0.305) |
| Rural percentage | 0.184  (0.236) | 0.138  (0.221) | 0.144  (0.207) | 0.239  (0.260) | 0.226  (0.240) |
| Married | 0.520  (0.500) | 0.508  (0.500) | 0.517  (0.500) | 0.520  (0.500) | 0.539  (0.498) |
| Dummies for number of children in home (none is omitted base category) | | | | | |
| One child | 0.158  (0.365) | 0.162  (0.369) | 0.158  (0.364) | 0.159  (0.366) | 0.154  (0.361) |
| Two children | 0.133  (0.339) | 0.136  (0.343) | 0.134  (0.341) | 0.129  (0.336) | 0.132  (0.338) |
| Three children | 0.052  (0.223) | 0.058  (0.233) | 0.049  (0.216) | 0.053  (0.225) | 0.052  (0.221) |
| Four children | 0.015  (0.123) | 0.017  (0.130) | 0.013  (0.115) | 0.016  (0.125) | 0.016  (0.127) |
| Five children or more | 0.006  (0.077) | 0.007  (0.082) | 0.006  (0.075) | 0.006  (0.077) | 0.006  (0.080) |
| *Economic controls* |  |  |  |  |  |
| Education dummies (less than high school degree is omitted base category) | | | | | |
| High school degree | 0.268  (0.443) | 0.252  (0.434) | 0.254  (0.435) | 0.288  (0.453) | 0.282  (0.450) |
| Some College | 0.329  (0.470) | 0.336  (0.472) | 0.322  (0.467) | 0.333  (0.471) | 0.330  (0.470) |
| College graduate | 0.286  (0.452) | 0.256  (0.436) | 0.333  (0.471) | 0.242  (0.428) | 0.302  (0.459) |
| Unemployed | 0.069  (0.253) | 0.079  (0.270) | 0.067  (0.250) | 0.069  (0.254) | 0.059  (0.236) |
| State unemployment rate | 8.161  (1.593) | 9.479  (1.426) | 8.095  (1.503) | 7.899  (1.396) | 7.228  (1.302) |
| Student | 0.110  (0.313) | 0.114  (0.318) | 0.110  (0.313) | 0.106  (0.308) | 0.112  (0.315) |
| Income dummies (Relative to the Federal Poverty Line)^a^ | | | | | |
| Income <100FPL | 0.144  (0.351) | 0.160  (0.367) | 0.125  (0.331) | 0.163  (0.369) | 0.130  (0.336) |
| 100FPL≤  Income<200FPL | 0.171  (0.376) | 0.193  (0.395) | 0.143  (0.350) | 0.199  (0.399) | 0.155  (0.362) |
| 200FPL≤  Income<300FPL | 0.162  (0.368) | 0.167  (0.372) | 0.147  (0.354) | 0.174  (0.379) | 0.164  (0.370) |
| 300FPL≤  Income<400FPL | 0.136  (0.343) | 0.130  (0.337) | 0.135  (0.341) | 0.136  (0.342) | 0.147  (0.354) |
| 400FPL≤  Income<500FPL | 0.106  (0.308) | 0.097  (0.296) | 0.112  (0.315) | 0.099  (0.299) | 0.117  (0.321) |
| Income ≥500FPL | 0.282  (0.450) | 0.253  (0.435) | 0.339  (0.473) | 0.229  (0.420) | 0.287  (0.452) |
| *Exchange controls* |  |  |  |  |  |
| State set up own exchange | 0.344  (0.475) | 0.631  (0.483) | 0.640  (0.480) | N/A | N/A |
| State exchange had glitches | 0.084  (0.277) | 0.073  (0.260) | 0.201  (0.401) | N/A | N/A |

Notes: Standard deviations are in parentheses. ­­

^a^Our baseline model includes dummy variables for each year of age as well as dummy variables for each 10-point increment of the FPL (50 total dummies). To conserve space, we present our descriptive statistics for age and income in broader categories in this table.

**Table A2. Implied Effects of the ACA at Low and High Mean Pre-Treatment Uninsured Rates for Full Sample and Income and Race Subsamples**

|  | Any insurance | | Any private | Employer-sponsored | Individually purchased | Medicaid | Other |
| --- | --- | --- | --- | --- | --- | --- | --- |
| **PANEL I: FULL SAMPLE** | | | | | | | |
| *Non-elderly adults aged 19-64 (sample size = 10,537,667)* | | | | | | | |
| *At Pre-Treatment Low Unin. Rate = 0.152* | | | | | | | |
| Medicaid  Expansion | 0.038***  (0.008) | | -0.007  (0.007) | 0.003  (0.005) | -0.008  (0.009) | 0.046***  (0.006) | 0.001  (0.001) |
|  |  | |  |  |  |  |  |
| Full ACA (w/  Medicaid) | 0.065***  (0.004) | | 0.021***  (0.009) | 0.013***  (0.003) | 0.009***  (0.002) | 0.046***  (0.005) | 0.001  (0.001) |
|  |  | |  |  |  |  |  |
| *At Pre-Treatment High Uninsured Rate = 0.259* | | | | | | | |
| Medicaid  Expansion | 0.064***  (0.014) | | -0.011  (0.012) | 0.004  (0.009) | -0.013  (0.015) | 0.079***  (0.011) | 0.001  (0.002) |
|  |  | |  |  |  |  |  |
| Full ACA (w/  Medicaid) | 0.110***  (0.007) | | 0.036***  (0.007) | 0.021***  (0.006) | 0.015***  (0.003) | 0.079***  (0.009) | 0.001  (0.002) |
|  |  | |  |  |  |  |  |
| **PANEL II: INCOME SUBSAMPLES** | | | | | | | |
| *Under 138% FPL (sample size = 1,949,375)* | | | | | | | |
| *At Pre-Treatment Low Unin. Rate = 0.330* | | | | | | | |
| Medicaid  Expansion | 0.142***  (0.027) | | -0.026  (0.015) | -0.006  (0.013) | -0020  (0.016) | 0.180***  (0.030) | -0.003  (0.003) |
|  |  | |  |  |  |  |  |
| Full ACA (w/  Medicaid) | 0.141***  (0.024) | | 0.015  (0.009) | 0.013  (0.007) | 0.002  (0.006) | 0.129***  (0.026) | 0.002  (0.002) |
|  |  | |  |  |  |  |  |
| *At Pre-Treatment High Uninsured Rate = 0.463* | | | | | | | |
| Medicaid  Expansion | 0.199***  (0.038) | | -0.036  (0.021) | -0.008  (0.018) | -0.028  (0.022) | 0.253***  (0.043) | -0.004  (0.004) |
|  |  | |  |  |  |  |  |
| Full ACA (w/  Medicaid) | 0.198***  (0.035) | | 0.021  (0.013) | 0.018  (0.010) | 0.002  (0.008) | 0.180***  (0.037) | 0.002  (0.003) |
|  |  | |  |  |  |  |  |
| *138%-400% FPL (sample size = 4,137,149)* | | | | | | | |
| *At Pre-Treatment Low Unin. Rate = 0.195* | | | | | | | |
| Medicaid  Expansion | 0.046***  (0.013) | | 0.009  (0.012) | 0.019  (0.013) | -0.007  (0.013) | 0.039**  (0.012) | 0.001  (0.002) |
|  |  | |  |  |  |  |  |
| Full ACA (w/  Medicaid) | 0.086***  (0.010) | | 0.055***  (0.011) | 0.041***  (0.009) | 0.016***  (0.005) | 0.035**  (0.011) | -0.001  (0.001) |
|  |  | |  |  |  |  |  |
| *At Pre-Treatment High Uninsured Rate = 0.296* | | | | | | | |
| Medicaid  Expansion | 0.070***  (0.020) | | 0.014  (0.019) | 0.028  (0.020) | -0.010  (0.020) | 0.059**  (0.019) | 0.001  (0.003) |
|  |  | |  |  |  |  |  |
| Full ACA (w/  Medicaid) | 0.131***  (0.016) | | 0.083***  (0.017) | 0.062***  (0.013) | 0.024***  (0.007) | 0.053**  (0.015) | -0.001  (0.002) |
|  |  | |  |  |  |  |  |
| *Over 400% FPL (sample size =* *4,482,022)* | | | | | | | |
| *At Pre-Treatment Low Unin. Rate = 0.054* | | | | | | | |
| Medicaid  Expansion | 0.009**  (0.003) | | 0.002  (0.005) | 0.005  (0.007) | -0.002  (0.006) | 0.008*  (0.004) | -0.001  (0.002) |
|  |  | |  |  |  |  |  |
| Full ACA (w/  Medicaid) | 0.023***  (0.003) | | 0.015***  (0.004) | 0.007  (0.004) | 0.008***  (0.002) | 0.007*  (0.003) | 0.001  (0.002) |
|  |  | |  |  |  |  |  |
| *At Pre-Treatment High Uninsured Rate = 0.088* | | | | | | | |
| Medicaid  Expansion | 0.015**  (0.005) | | 0.003  (0.007) | 0.008  (0.012) | -0.003  (010) | 0.014*  (0.006) | -0.002  (0.003) |
| Full ACA (w/  Medicaid) | 0.038***  (0.004) | | 0.025***  (0.007) | 0.011  (0.007) | 0.014***  (0.004) | 0.012*  (0.006) | 0.001  (0.003) |
|  |  | |  |  |  |  |  |
|  | | | | | | | |
| **PANEL III: RACE / ETHNICITY SUBSAMPLES** | | | | | | | |
| *Non-Hispanic White (sample size = 7,149,482)* | | | | | | | |
| *At Pre-Treatment Low Unin. Rate = 0.108* | | | | | | | |
| Medicaid  Expansion | | 0.038***  (0.004) | -0.002  (0.004) | 0.003  (0.006) | -0.003  (0.005) | 0.043***  (0.006) | -0.001  (0.001) |
|  | |  |  |  |  |  |  |
| Full ACA (w/  Medicaid) | | 0.058***  (0.004) | 0.014***  (0.003) | 0.008**  (0.003) | 0.005*  (0.002) | 0.050***  (0.005) | -0.002***  (0.001) |
|  | |  |  |  |  |  |  |
| *At Pre-Treatment High Uninsured Rate = 0.187* | | | | | | | |
| Medicaid  Expansion | | 0.065***  (0.007) | -0.003  (0.007) | 0.005  (0.010) | -0.005  (0.009) | 0.075***  (0.011) | -0.0003  (0.002) |
|  | |  |  |  |  |  |  |
| Full ACA (w/  Medicaid) | | 0.100***  (0.007) | 0.025***  (0.006) | 0.014**  (0.005) | 0.009*  (0.004) | 0.087***  (0.009) | -0.004***  (0.001) |
|  | |  |  |  |  |  |  |
| *Non-White (sample size = 3,388,185)* | | | | | | | |
| *At Pre-Treatment Low Unin. Rate = 0.233* | | | | | | | |
| Medicaid  Expansion | | 0.054***  (0.015) | -0.011  (0.015) | 0.004  (0.007) | -0.011  (0.015) | 0.064***  (0.010) | 0.004*  (0.002) |
|  | |  |  |  |  |  |  |
| Full ACA (w/  Medicaid) | | 0.088***  (0.007) | 0.030***  (0.007) | 0.020**  (0.006) | 0.014**  (0.004) | 0.058***  (0.009) | 0.003*  (0.002) |
| *At Pre-Treatment High Uninsured Rate = 0.363* | | | | | | | |
| Medicaid  Expansion | | 0.084***  (0.024) | -0.016  (0.023) | 0.006  (0.011) | -0.017  (0.024) | 0.100*** (0.015) | 0.007*  (0.003) |
|  | |  |  |  |  |  |  |
| Full ACA (w/  Medicaid) | | 0.138***  (0.011) | 0.047***  (0.010) | 0.030**  (0.009) | 0.021**  (0.007) | 0.090***  (0.015) | 0.005*  (0.002) |

Notes: Results are effects of the ACA on the proportion of residents with the specified type of insurance, evaluated at the means of the lower and upper halves of the pre-treatment uninsured rate distribution. Standard errors, heteroscedasticity-robust and clustered by state, are in parentheses. *** indicates statistically significant at 0.1% level; ** 1% level; * 5% level. Sampling weights are used. All regressions include area and time fixed effects and the full set of controls.

**Table A3. Implied Effects of the ACA at Low and High Mean Pre-Treatment Uninsured Rates for Marital Status and Age Subsamples**

|  | Any insurance | | Any private | Employer-sponsored | Individually purchased | Medicaid | Other |
| --- | --- | --- | --- | --- | --- | --- | --- |
| **PANEL I: MARITAL STATUS SUBSAMPLES** | | | | | | | |
| *Married (sample size =5,978,285)* | | | | | | | |
| *At Pre-Treatment Low Unin. Rate = 0.099* | | | | | | | |
| Medicaid  Expansion | 0.022***  (0.006) | | -0.004  (0.04) | 0.001  (0.003) | -0.004  (0.006) | 0.027***  (0.003) | 0.001  (0.001) |
|  |  | |  |  |  |  |  |
| Full ACA (w/  Medicaid) | 0.042***  (0.002) | | 0.015***  (0.002) | 0.007***  (0.002) | 0.008***  (0.002) | 0.028***  (0.002) | 0.001  (0.001) |
|  |  | |  |  |  |  |  |
| *At Pre-Treatment High Uninsured Rate = 0.203* | | | | | | | |
| Medicaid  Expansion | 0.045***  (0.011) | | -0.008  (0.009) | 0.001  (0.006) | -0.008  (0.011) | 0.055***  (0.007) | 0.001  (0.002) |
|  |  | |  |  |  |  |  |
| Full ACA (w/  Medicaid) | 0.086***  (0.005) | | 0.031***  (0.004) | 0.014***  (0.004) | 0.017***  (0.003) | 0.058***  (0.005) | 0.001  (0.002) |
|  |  | |  |  |  |  |  |
| *Unmarried (sample size =4,559,382)* | | | | | | | |
| *At Pre-Treatment Low Unin. Rate = 0.213* | | | | | | | |
| Medicaid  Expansion | 0.063***  (0.010) | | -0.007  (0.010) | 0.004  (0.009) | -0.009  (0.012) | 0.073***  (0.010) | 0.001  (0.001) |
|  |  | |  |  |  |  |  |
| Full ACA (w/  Medicaid) | 0.094***  (0.007) | | 0.027***  (0.007) | 0.020***  (0.005) | 0.008*  (0.033) | 0.070***  (0.008) | 0.001  (0.001) |
|  |  | |  |  |  |  |  |
| *At Pre-Treatment High Uninsured Rate = 0.333* | | | | | | | |
| Medicaid  Expansion | 0.098***  (0.016) | | -0.011  (0.016) | 0.006  (0.014) | -0.014  (0.019) | 0.114***  (0.016) | 0.002  (0.002) |
|  |  | |  |  |  |  |  |
| Full ACA (w/  Medicaid) | 0.147***  (0.011) | | 0.042***  (0.011) | 0.031***  (0.007) | 0.013*  (0.005) | 0.110***  (0.013) | 0.001  (0.002) |
|  |  | |  |  |  |  |  |
|  | | | | | | | |
| **PANEL II: AGE SUBSAMPLES** | | | | | | | |
| *Ages 19-26 (sample size=1,562,121)* | | | | | | | |
| *At Pre-Treatment Low Unin. Rate = 0.204* | | | | | | | |
| Medicaid  Expansion | | 0.058***  (0.011) | -0.006  (0.011) | 0.005  (0.008) | -0.008  (0.009) | 0.068***  (0.010) | 0.001  (0.002) |
|  | |  |  |  |  |  |  |
| Full ACA (w/  Medicaid) | | 0.094***  (0.007) | 0.023**  (0.007) | 0.018**  (0.006) | 0.007*  (0.003) | 0.073***  (0.009) | 0.003*  (0.001) |
|  | |  |  |  |  |  |  |
| *At Pre-Treatment High Uninsured Rate = 0.348* | | | | | | | |
| Medicaid  Expansion | | 0.098***  (0.019) | -0.010  (0.019) | 0.008  (0.014) | -0.014  (0.015) | 0.116***  (0.017) | 0.002  (0.003) |
|  | |  |  |  |  |  |  |
| Full ACA (w/  Medicaid) | | 0.159***  (0.011) | 0.040**  (0.012) | 0.031**  (0.011) | 0.011*  (0.005) | 0.125***  (0.016) | 0.005*  (0.002) |
|  | |  |  |  |  |  |  |
| *Ages 27-34 (sample size=1,667,573)* | | | | | | | |
| *At Pre-Treatment Low Unin. Rate = 0.193* | | | | | | | |
| Medicaid  Expansion | | 0.041***  (0.009) | -0.009  (0.008) | -0.004  (0.006) | -0.003  (0.006) | 0.052***  (0.010) | 0.001  (0.002) |
|  | |  |  |  |  |  |  |
| Full ACA (w/  Medicaid) | | 0.069***  (0.007) | 0.015*  (0.006) | 0.008  (0.005) | 0.007*  (0.003) | 0.056***  (0.007) | 0.002  (0.002) |
| *At Pre-Treatment High Uninsured Rate = 0.319* | | | | | | | |
| Medicaid  Expansion | | 0.067***  (0.015) | -0.015  (0.013) | -0.007  (0.010) | -0.005  (0.011) | 0.087***  (0.016) | 0.002  (0.004) |
|  | |  |  |  |  |  |  |
| Full ACA (w/  Medicaid) | | 0.114***  (0.011) | 0.025*  (0.010) | 0.013  (0.008) | 0.012*  (0.005) | 0.093***  (0.011) | 0.003  (0.003) |
| *Ages 35-49 (sample size=3,330,941)* | | | | | | | |
| *At Pre-Treatment Low Unin. Rate = 0.151* | | | | | | | |
| Medicaid  Expansion | | 0.026***  (0.008) | -0.009  (0.007) | -0.001  (0.005) | -0.006  (0.008) | 0.035***  (0.006) | 0.002  (0.001) |
|  | |  |  |  |  |  |  |
| Full ACA (w/  Medicaid) | | 0.055***  (0.004) | 0.020***  (0.004) | 0.013***  (0.003) | 0.007***  (0.002) | 0.036***  (0.005) | 0.001  (0.001) |
| *At Pre-Treatment High Uninsured Rate = 0.267* | | | | | | | |
| Medicaid  Expansion | | 0.046***  (0.013) | -0.015  (0.012) | -0.003  (0.008) | -0.011  (0.014) | 0.061***  (0.011) | 0.003  (0.002) |
|  | |  |  |  |  |  |  |
| Full ACA (w/  Medicaid) | | 0.096***  (0.006) | 0.035***  (0.007) | 0.022***  (0.006) | 0.013***  (0.004) | 0.063***  (0.008) | 0.002  (0.002) |
| *Ages 50-64 (sample size=3,977,032)* | | | | | | | |
| *At Pre-Treatment Low Unin. Rate = 0.108* | | | | | | | |
| Medicaid  Expansion | | 0.027***  (0.006) | -0.003  (0.004) | 0.006  (0.006) | -0.007  (0.009) | 0.033***  (0.003) | -0.001  (0.002) |
|  | |  |  |  |  |  |  |
| Full ACA (w/  Medicaid) | | 0.052***  (0.003) | 0.023***  (0.003) | 0.011***  (0.002) | 0.012***  (0.002) | 0.033***  (0.002) | -0.002  (0.001) |
| *At Pre-Treatment High Uninsured Rate = 0.195* | | | | | | | |
| Medicaid  Expansion | | 0.049***  (0.010) | -0.006  (0.007) | 0.011  (0.011) | -0.013  (0.016) | 0.059***  (0.006) | -0.003  (0.003) |
|  | |  |  |  |  |  |  |
| Full ACA (w/  Medicaid) | | 0.094***  (0.006) | 0.041***  (0.005) | 0.021***  (0.004) | 0.022***  (0.004) | 0.059***  (0.004) | -0.003  (0.002) |

Notes: Results are effects of the ACA on the proportion of residents with the specified type of insurance, evaluated at the means of the lower and upper halves of the pre-treatment uninsured rate distribution. Standard errors, heteroscedasticity-robust and clustered by state, are in parentheses. *** indicates statistically significant at 0.1% level; ** 1% level; * 5% level. Sampling weights are used. All regressions include area and time fixed effects and the full set of controls.

**Table A4. Implied Effects of the ACA at Low and High Mean Pre-Treatment Uninsured Rates for Gender and Rural/Urban Subsamples**

|  | Any insurance | | Any private | Employer-sponsored | Individually purchased | Medicaid | Other |
| --- | --- | --- | --- | --- | --- | --- | --- |
| **PANEL I: GENDER SUBSAMPLES** | | | | | | | |
| *Women (sample size =5,473,836)* | | | | | | | |
| *At Pre-Treatment Low Unin. Rate =0.140* | | | | | | | |
| Medicaid  Expansion | 0.048***  (0.008) | | -0.004  (0.007) | 0.005  (0.008) | -0.008  (0.008) | 0.054***  (0.010) | 0.001  (0.001) |
|  |  | |  |  |  |  |  |
| Full ACA (w/  Medicaid) | 0.072***  (0.006) | | 0.023***  (0.006) | 0.017***  (0.004) | 0.006*  (0.003) | 0.051***  (0.008) | 0.001  (0.001) |
|  |  | |  |  |  |  |  |
| *At Pre-Treatment High Uninsured Rate = 0.236* | | | | | | | |
| Medicaid  Expansion | 0.081***  (0.014) | | -0.007  (0.011) | 0.009  (0.013) | -0.014  (0.013) | 0.091***  (0.017) | 0.002  (0.002) |
|  |  | |  |  |  |  |  |
| Full ACA (w/  Medicaid) | 0.122***  (0.010) | | 0.038***  (0.009) | 0.029***  (0.007) | 0.010*  (0.005) | 0.085***  (0.014) | 0.002  (0.001) |
|  |  | |  |  |  |  |  |
| *Men (sample size =5,094,710)* | | | | | | | |
| *At Pre-Treatment Low Unin. Rate = 0.178* | | | | | | | |
| Medicaid  Expansion | 0.045***  (0.009) | | -0.003  (0.008) | 0.008  (0.009) | -0.006  (0.012) | 0.053***  (0.009) | -0.002  (0.002 |
|  |  | |  |  |  |  |  |
| Full ACA (w/  Medicaid) | 0.077***  (0.006) | | 0.032***  (0.007) | 0.022***  (0.004) | 0.013***  (0.004) | 0.050***  (0.008) | -0.001  (0.002) |
|  |  | |  |  |  |  |  |
| *At Pre-Treatment High Uninsured Rate = 0.275* | | | | | | | |
| Medicaid  Expansion | 0.070***  (0.014) | | -0.004  (0.012) | 0.013  (0.013) | -0.010  (0.018) | 0.082***  (0.014) | -0.003  (0.003) |
|  |  | |  |  |  |  |  |
| Full ACA (w/  Medicaid) | 0.119***  (0.009) | | 0.049***  (0.010) | 0.034***  (0.007) | 0.020***  (0.005) | 0.077***  (0.013) | -0.002  (0.002) |
|  |  | |  |  |  |  |  |
|  | | | | | | | |
| **PANEL II: RURAL vs. URBAN SUBSAMPLES** | | | | | | | |
| *Rural (sample size=1,964,610)* | | | | | | | |
| *At Pre-Treatment Low Unin. Rate = 0.163* | | | | | | | |
| Medicaid  Expansion | | 0.062***  (0.012) | -0.012  (0.010) | -0.009  (0.006) | -0.003  (0.009) | 0.076***  (0.015) | 0.001  (0.003) |
|  | |  |  |  |  |  |  |
| Full ACA (w/  Medicaid) | | 0.092***  (0.019) | 0.014  (0.008) | 0.011** (0.004) | -0.003  (0.007) | 0.085***  (0.013) | -0.003  (0.002) |
|  | |  |  |  |  |  |  |
| *At Pre-Treatment High Uninsured Rate = 0.254* | | | | | | | |
| Medicaid  Expansion | | 0.097***  (0.019) | -0.019  (0.016) | -0.014  (0.010) | -0.004  (0.014) | 0.118***  (0.013) | 0.001  (0.005) |
|  | |  |  |  |  |  |  |
| Full ACA (w/  Medicaid) | | 0.144***  (0.017) | 0.022  (0.013) | 0.017**  (0.006) | -0.004  (0.012) | 0.133***  (0.024) | -0.005  (0.003) |
|  | |  |  |  |  |  |  |
| *Urban (sample size=8,603,936)* | | | | | | | |
| *At Pre-Treatment Low Unin. Rate = 0.158* | | | | | | | |
| Medicaid  Expansion | | 0.044***  (0.009) | -0.003  (0.007) | 0.009  (0.009) | -0.008  (0.010) | 0.050***  (0.009) | -0.001  (0.001) |
|  | |  |  |  |  |  |  |
| Full ACA (w/  Medicaid) | | 0.071***  (0.006) | 0.028***  (0.006) | 0.020***  (0.004) | 0.010***  (0.003) | 0.045***  (0.008) | 0.001  (0.001) |
| *At Pre-Treatment High Uninsured Rate = 0.257* | | | | | | | |
| Medicaid  Expansion | | 0.071***  (0.014) | -0.004  (0.012) | 0.014  (0.014) | -0.013  (0.016) | 0.081***  (0.015) | -0.001  (0.002) |
|  | |  |  |  |  |  |  |
| Full ACA (w/  Medicaid) | | 0.115***  (0.010) | 0.046***  (0.0100 | 0.032***  (0.007) | 0.017***  (0.005) | 0.074***  (0.012) | 0.001 |

Notes: Results are effects of the ACA on the proportion of residents with the specified type of insurance, evaluated at the means of the lower and upper halves of the pre-treatment uninsured rate distribution. Standard errors, heteroscedasticity-robust and clustered by state, are in parentheses. *** indicates statistically significant at 0.1% level; ** 1% level; * 5% level. Sampling weights are used. All regressions include area and time fixed effects and the full set of controls.

**Table A5. Event Study Results for Full Sample and Income and Race Subsamples**

|  | Any insurance | | Any private | Employer-sponsored | Individually purchased | Medicaid | Other |
| --- | --- | --- | --- | --- | --- | --- | --- |
| **PANEL I: FULL SAMPLE** | | | | | | | |
| *Non-elderly adults aged 19-64 (pre-treatment uninsured rate=0.203, sample size=10,537,667)* | | | | | | | |
| Unin. Rate*  Med. Exp.*2011 | 0.018  (0.030) | | -0.031  (0.033) | -0.041  (0.036) | -0.020  (0.021) | 0.047  (0.031) | -0.000  (0.009) |
|  |  | |  |  |  |  |  |
| Unin. Rate*  Med. Exp.*2012 | -0.013  (0.037) | | -0.004  (0.033) | -0.017  (0.027) | -0.017  (0.025) | -0.004  (0.015) | -0.007  (0.006) |
|  |  | |  |  |  |  |  |
| Uninsured  Rate*2011 | 0.011  (0.020) | | 0.039  (0.030) | 0.026  (0.032) | 0.032*  (0.016) | -0.023  (0.019) | -0.001  (0.007) |
|  |  | |  |  |  |  |  |
| Uninsured  Rate*2012 | 0.030  (0.028) | | 0.034  (0.027) | 0.012  (0.021) | 0.046***  (0.013) | 0.001  (0.007) | 0.000  (0.003) |
|  |  | |  |  |  |  |  |
| **PANEL II: INCOME SUBSAMPLES** | | | | | | | |
| *Under 138% FPL (Pre-Treatment Uninsured Rate = 0.395, sample size = 1,949,375)* | | | | | | | |
| Unin. Rate*  Med. Exp.*2011 | 0.100*  (0.049) | | -0.058  (0.048) | -0.105**  (0.036) | 0.029  (0.037) | 0.162**  (0.056) | -0.004  (0.011) |
|  |  | |  |  |  |  |  |
| Unin. Rate*  Med. Exp.*2012 | 0.064  (0.044) | | 0.020  (0.312) | 0.022  (0.023) | -0.009  (0.026) | 0.019  (0.036) | -0.008  (0.013) |
|  |  | |  |  |  |  |  |
| Uninsured  Rate*2011 | -0.013  (0.018) | | 0.062  (0.042) | 0.066  (0.033) | -0.001  (0.033) | -0.016  (0.040) | -0.001  (0.011) |
|  |  | |  |  |  |  |  |
| Uninsured  Rate*2012 | -0.025  (0.027) | | -0.004  (0.025) | -0.038*  (0.016) | 0.040*  (0.019) | -0.004  (0.013) | -0.015  (0.008) |
|  |  | |  |  |  |  |  |
| *138%-400% FPL (Pre-Treatment Uninsured Rate = 0.237, sample size = 4,137,149)* | | | | | | | |
| Unin. Rate*  Med. Exp.*2011 | -0.017  (0.032) | | -0.040  (0.051) | -0.054  (0.050) | -0.025  (0.034) | 0.018  (0.030) | -0.000  (0.012) |
|  |  | |  |  |  |  |  |
| Unin. Rate*  Med. Exp.*2012 | -0.065  (0.043) | | -0.038  (0.055) | -0.058  (0.049) | -0.012  (0.027) | -0.020  (0.027) | 0.006  (0.004) |
|  |  | |  |  |  |  |  |
| Uninsured  Rate*2011 | 0.005  (0.028) | | 0.027  (0.043) | 0.018  (0.030) | 0.025  (0.025) | -0.008  (0.022) | -0.007  (0.010) |
|  |  | |  |  |  |  |  |
| Uninsured  Rate*2012 | 0.022  (0.040) | | 0.021  (0.046) | 0.022  (0.034) | 0.018  (0.021) | 0.005  (0.012) | 0.006  (0.004) |
|  |  | |  |  |  |  |  |
|  |  | |  |  |  |  |  |
| *Over 400% FPL (Pre-Treatment Uninsured Rate = 0.067, sample size =* *4,482,022)* | | | | | | | |
| Unin. Rate*  Med. Exp.*2011 | -0.030  (0.062) | | -0.109  (0.099) | -0.168  (0.127) | -0.007  (0.070) | 0.064  (0.034) | 0.031  (0.042) |
|  |  | |  |  |  |  |  |
| Unin. Rate*  Med. Exp.*2012 | 0.070  (0.051) | | 0.077  (0.066) | 0.001  (0.085) | -0.017  (0.097) | -0.003  (0.044) | 0.010  (0.032) |
|  |  | |  |  |  |  |  |
| Uninsured  Rate*2011 | 0.093  (0.049) | | 0.124  (0.074) | 0.119  (0.102) | 0.070  (0.051) | -0.003  (0.019) | -0.024  (0.024) |
|  |  | |  |  |  |  |  |
| Uninsured  Rate*2012 | 0.047  (0.040) | | 0.044  (0.052) | -0.021  (0.062) | 0.132*  (0.060) | 0.026  (0.019) | -0.014  (0.020) |
|  | | | | | | | |
| **PANEL III: RACE / ETHNICITY SUBSAMPLES** | | | | | | | |
| *Non-Hispanic White (Pre-Treatment Uninsured Rate = 0.144, sample size = 7,149,482)* | | | | | | | |
| Unin. Rate*  Med. Exp.*2011 | | -0.013  (0.042) | -0.054  (0.039) | -0.053  (0.042) | -0.048  (0.033) | 0.043  (0.034) | -0.007  (0.017) |
|  | |  |  |  |  |  |  |
| Unin. Rate*  Med. Exp.*2012 | | -0.009  (0.049) | 0.020  (0.044) | -0.027  (0.040) | 0.011  (0.031) | -0.016  (0.024) | -0.007  (0.013) |
|  | |  |  |  |  |  |  |
| Uninsured  Rate*2011 | | 0.066*  (0.028) | 0.086**  (0.030) | 0.061  (0.036) | 0.074***  (0.024) | -0.019  (0.023) | 0.001  (0.014) |
|  | |  |  |  |  |  |  |
| Uninsured  Rate*2012 | | 0.075  (0.038) | 0.065  (0.047) | 0.045  (0.034) | 0.068***  (0.014) | 0.021  (0.014) | -0.005  (0.010) |
|  | |  |  |  |  |  |  |
| *Non-White (Pre-Treatment Uninsured Rate = 0.306, sample size = 3,388,185)* | | | | | | | |
| Unin. Rate*  Med. Exp.*2011 | | -0.013  (0.052) | -0.082  (0.051) | -0.102  (0.051) | -0.023  (0.031) | 0.054  (0.027) | 0.017  (0.015) |
|  | |  |  |  |  |  |  |
| Unin. Rate*  Med. Exp.*2012 | | -0.031  (0.055) | -0.027  (0.046) | -0.036  (0.043) | -0.001  (0.023) | 0.008  (0.025) | -0.012  (0.010) |
|  | |  |  |  |  |  |  |
| Uninsured  Rate*2011 | | 0.093*  (0.040) | 0.113*  (0.045) | 0.108*  (0.043) | 0.015  (0.014) | -0.011  (0.020) | -0.007  (0.014) |
|  | |  |  |  |  |  |  |
| Uninsured  Rate*2012 | | 0.079  (0.046) | 0.066  (0.042) | 0.062  (0.038) | 0.002  (0.018) | 0.004  (0.014) | 0.010  (0.009) |

Notes: Coefficient estimates are shown. Standard errors, heteroscedasticity-robust and clustered by state, are in parentheses. *** indicates statistically significant at 0.1% level; ** 1% level; * 5% level. Sampling weights are used. All regressions include area and time fixed effects, the full set of controls, Medicaid Expansion*2011, and Medicaid Expansion*2012.

**Table A6. Event Study Results for Marital Status and Age Subsamples**

|  | Any insurance | Any private | Employer-sponsored | Individually purchased | Medicaid | Other |
| --- | --- | --- | --- | --- | --- | --- |
| **PANEL I: MARITAL STATUS SUBSAMPLES** | | | | | | |
| *Married (pre-treatment uninsured rate = 0.141, sample size = 5,978,285)* | | | | | | |
| Unin. Rate*  Med. Exp.*2011 | -0.016  (0.033) | -0.016  (0.035) | -0.029  (0.034) | -0.009  (0.024) | 0.009  (0.023) | -0.012  (0.013) |
|  |  |  |  |  |  |  |
| Unin. Rate*  Med. Exp.*2012 | -0.010  (0.033) | 0.003  (0.039) | -0.012  (0.026) | -0.006  (0.035) | 0.005  (0.019) | -0.017  (0.010) |
|  |  |  |  |  |  |  |
| Uninsured  Rate*2011 | 0.047*  (0.020) | 0.033  (0.027) | 0.019  (0.025) | 0.031  (0.019) | -0.001  (0.019) | 0.017  (0.010) |
|  |  |  |  |  |  |  |
| Uninsured  Rate*2012 | 0.040  (0.025) | 0.036  (0.026) | 0.015  (0.013) | 0.041  (0.023) | -0.001  (0.007) | 0.008  (0.007) |
|  |  |  |  |  |  |  |
| *Unmarried (pre-treatment uninsured rate = 0.272, sample size= 4,559,382)* | | | | | | |
| Unin. Rate*  Med. Exp.*2011 | 0.032  (0.045) | -0.048  (0.042) | -0.052  (0.047) | -0.027  (0.029) | 0.072  (0.040) | 0.006  (0.010) |
|  |  |  |  |  |  |  |
| Unin. Rate*  Med. Exp.*2012 | -0.027  (0.052) | -0.022  (0.038) | -0.037  (0.041) | -0.020  (0.021) | -0.008  (0.025) | 0.001  (0.009) |
|  |  |  |  |  |  |  |
| Uninsured  Rate*2011 | 0.037  (0.030) | 0.075*  (0.035) | 0.060  (0.043) | 0.032  (0.024) | -0.020  (0.020) | -0.010  (0.008) |
|  |  |  |  |  |  |  |
| Uninsured  Rate*2012 | 0.074  (0.040) | 0.059  (0.032) | 0.043  (0.036) | 0.042***  (0.012) | 0.025  (0.013) | -0.004  (0.006) |
|  |  |  |  |  |  |  |
| **PANEL II: AGE SUBSAMPLES** | | | | | | |
| *Ages 19-26 (Pre-Treatment Uninsured Rate = 0.270, sample size =1,562,121)* | | | | | | |
| Unin. Rate*  Med. Exp.*2011 | -0.031  (0.060) | -0.086  (0.049) | -0.061  (0.051) | -0.060*  (0.026) | 0.039  (0.043) | 0.007  (0.011) |
|  |  |  |  |  |  |  |
| Unin. Rate*  Med. Exp.*2012 | 0.038  (0.056) | 0.009  (0.048) | 0.007  (0.048) | -0.009  (0.034) | -0.007  (0.033) | 0.021  (0.013) |
|  |  |  |  |  |  |  |
| Uninsured  Rate*2011 | 0.142**  (0.044) | 0.129**  (0.042) | 0.104*  (0.044) | 0.031  (0.021) | 0.020  (0.015) | -0.001  (0.009) |
|  |  |  |  |  |  |  |
| Uninsured  Rate*2012 | 0.091**  (0.032) | 0.078*  (0.037) | 0.043  (0.039) | 0.046*  (0.020) | 0.031  (0.018) | -0.008  (0.008) |
|  |  |  |  |  |  |  |
| *Ages 27-34 (Pre-Treatment Uninsured Rate = 0.256, sample size =1,667,573)* | | | | | | |
| Unin. Rate*  Med. Exp.*2011 | 0.023  (0.064) | -0.020  (0.056) | -0.021  (0.062) | -0.030  (0.034) | 0.046  (0.028) | -0.001  (0.015) |
|  |  |  |  |  |  |  |
| Unin. Rate*  Med. Exp.*2012 | -0.059  (0.067) | -0.056  (0.052) | -0.023  (0.049) | -0.073**  (0.023) | 0.024  (0.035) | -0.025  (0.013) |
|  |  |  |  |  |  |  |
| Uninsured  Rate*2011 | 0.087  (0.054) | 0.089  (0.053) | 0.088  (0.057) | 0.031  (0.027) | -0.003  (0.022) | 0.010  (0.011) |
|  |  |  |  |  |  |  |
| Uninsured  Rate*2012 | 0.172**  (0.053) | 0.137**  (0.045) | 0.104*  (0.041) | 0.053***  (0.016) | 0.021  (0.022) | 0.020*  (0.008) |
|  |  |  |  |  |  |  |
| *Ages 35-49 (Pre-Treatment Uninsured Rate = 0.201, sample size = 3,330,941)* | | | | | | |
| Unin. Rate*  Med. Exp.*2011 | 0.031  (0.040) | -0.025  (0.032) | -0.053  (0.040) | -0.001  (0.025) | 0.070*  (0.033) | -0.004  (0.017) |
|  |  |  |  |  |  |  |
| Unin. Rate*  Med. Exp.*2012 | 0.017  (0.054) | 0.010  (0.044) | -0.016  (0.033) | -0.004  (0.027) | 0.035  (0.019) | -0.018  (0.017) |
|  |  |  |  |  |  |  |
| Uninsured  Rate*2011 | 0.055*  (0.023) | 0.073***  (0.021) | 0.061*  (0.026) | 0.036**  (0.013) | -0.027  (0.023) | 0.004  (0.015) |
|  |  |  |  |  |  |  |
| Uninsured  Rate*2012 | 0.064  (0.044) | 0.067*  (0.032) | 0.060*  (0.024) | 0.038*  (0.018) | -0.011  (0.010) | 0.010  (0.014) |
|  |  |  |  |  |  |  |
| *Ages 50-64 (Pre-Treatment Uninsured Rate = 0.145, sample size = 3,977,032)* | | | | | | |
| Unin. Rate*  Med. Exp.*2011 | 0.001  (0.050) | 0.002  (0.042) | -0.008  (0.034) | -0.013  (0.037) | 0.008  (0.031) | -0.019  (0.014) |
|  |  |  |  |  |  |  |
| Unin. Rate*  Med. Exp.*2012 | -0.078  (0.048) | -0.027  (0.044) | -0.047  (0.031) | -0.012  (0.043) | -0.050  (0.030) | -0.004  (0.013) |
|  |  |  |  |  |  |  |
| Uninsured  Rate*2011 | 0.093***  (0.028) | 0.059  (0.039) | 0.022  (0.033) | 0.059*  (0.028) | 0.033  (0.019) | 0.010  (0.009) |
|  |  |  |  |  |  |  |
| Uninsured  Rate*2012 | 0.116***  (0.027) | 0.078***  (0.022) | 0.034  (0.020) | 0.080***  (0.019) | 0.050  (0.18) | -0.008  (0.008) |

Notes: Coefficient estimates are shown. Standard errors, heteroscedasticity-robust and clustered by state, are in parentheses. *** indicates statistically significant at 0.1% level; ** 1% level; * 5% level. Sampling weights are used. All regressions include area and time fixed effects, the full set of controls, Medicaid Expansion*2011, and Medicaid Expansion*2012.

**Table A7. Event Study Results for Gender and Rural/Urban Subsamples**

|  | Any insurance | Any private | Employer-sponsored | Individually purchased | Medicaid | Other |
| --- | --- | --- | --- | --- | --- | --- |
| **PANEL I: GENDER SUBSAMPLES** | | | | | | |
| *Women (pre-treatment uninsured rate=0.186, sample size=5,473,836)* | | | | | | |
| Unin. Rate*  Med. Exp.*2011 | 0.029  (0.032) | -0.038  (0.046) | -0.066  (0.041) | -0.015  (0.036) | 0.080  (0.040) | -0.010  (0.010) |
|  |  |  |  |  |  |  |
| Unin. Rate*  Med. Exp.*2012 | -0.005  (0.038) | -0.002  (0.036) | -0.022  (0.032) | -0.017  (0.031) | -0.005  (0.023) | -0.007  (0.011) |
|  |  |  |  |  |  |  |
| Uninsured  Rate*2011 | -0.019  (0.020) | 0.029  (0.040) | 0.013  (0.027) | 0.034  (0.034) | -0.049  (0.025) | -0.001  (0.007) |
|  |  |  |  |  |  |  |
| Uninsured  Rate*2012 | -0.001  (0.028) | 0.009  (0.030) | -0.011  (0.020) | 0.045  (0.024) | 0.000  (0.009) | -0.004  (0.007) |
|  |  |  |  |  |  |  |
| *Men (pre-treatment uninsured rate=0.223, sample size=5,094,710)* | | | | | | |
| Unin. Rate*  Med. Exp.*2011 | -0.005  (0.031) | -0.081  (0.041) | -0.103*  (0.034) | -0.021  (0.032) | 0.071  (0.037) | 0.005  (0.013) |
|  |  |  |  |  |  |  |
| Unin. Rate*  Med. Exp.*2012 | -0.032  (0.032) | -0.020  (0.038) | -0.034  (0.039) | -0.021  (0.023) | -0.003  (0.024) | -0.005  (0.009) |
|  |  |  |  |  |  |  |
| Uninsured  Rate*2011 | -0.001  (0.027) | 0.044  (0.035) | 0.039  (0.037) | 0.022  (0.022) | -0.035  (0.021) | -0.004  (0.011) |
|  |  |  |  |  |  |  |
| Uninsured  Rate*2012 | 0.007  (0.030) | 0.020  (0.032) | 0.001  0.028 | 0.040***  (0.011) | -0.009  (0.015) | 0.002  (0.006) |
|  |  |  |  |  |  |  |
| **PANEL II: RURAL vs. URBAN SUBSAMPLES** | | | | | | |
| *Rural (Pre-Treatment Uninsured Rate=0.212, sample size=1,964,610)* | | | | | | |
| Unin. Rate*  Med. Exp.*2011 | 0.067  (0.031) | -0.011  (0.046) | 0.021  (0.063 | -0.017  (0.040) | 0.109*  (0.050) | -0.016  (0.033) |
|  |  |  |  |  |  |  |
| Unin. Rate*  Med. Exp.*2012 | 0.041  (0.069) | 0.061  (0.047) | 0.110  (0.058) | -0.089  (0.047) | -0.063  (0.060) | -0.024  (0.019) |
|  |  |  |  |  |  |  |
| Uninsured  Rate*2011 | -0.134***  (0.031) | -0.109*  (0.046) | -0.135**  (0.047) | 0.035  (0.031) | -0.031  (0.024) | 0.013  (0.027) |
|  |  |  |  |  |  |  |
| Uninsured  Rate*2012 | -0.048  (0.041) | -0.039  (0.042) | -0.066  (0.040) | 0.055*  (0.027) | 0.022  (0.030) | -0.024  (0.019) |
|  |  |  |  |  |  |  |
| *Urban (Pre-Treatment Uninsured Rate =0.203, sample size =8,603,936)* | | | | | | |
| Unin. Rate*  Med. Exp.*2011 | 0.018  (0.029) | -0.065  (0.041) | -0.097*  (0.041) | -0.020  (0.031) | 0.086*  (0.036) | -0.001  (0.010) |
|  |  |  |  |  |  |  |
| Unin. Rate*  Med. Exp.*2012 | -0.018  (0.035) | -0.015  (0.038) | -0.042  (0.036) | -0.010  (0.031) | 0.008  (0.018) | -0.012  (0.008) |
|  |  |  |  |  |  |  |
| Uninsured  Rate*2011 | 0.002  (0.022) | 0.049  (0.037) | 0.046  (0.034) | 0.024  (0.025) | -0.042*  (0.020) | -0.005  (0.008) |
|  |  |  |  |  |  |  |
| Uninsured  Rate*2012 | 0.007  (0.031) | 0.019  (0.033) | 0.002  (0.028) | 0.042*  (0.017) | -0.008  (0.009) | 0.001  (0.004) |

Notes: Coefficient estimates are shown. Standard errors, heteroscedasticity-robust and clustered by state, are in parentheses. *** indicates statistically significant at 0.1% level; ** 1% level; * 5% level. Sampling weights are used. All regressions include area and time fixed effects, the full set of controls, Medicaid Expansion*2011, and Medicaid Expansion*2012.

**Table A8. Implied Effects of the ACA at Mean Pre-Treatment Uninsured Rate for Full Sample**

|  | Income to poverty ratio | Probability of being married | Probability of getting married during last 12 months | Probability of getting divorced during last 12 months | Probability of getting married or divorced during last 12 months |
| --- | --- | --- | --- | --- | --- |
| *Non-elderly adults aged 19-64 (pre-treatment uninsured rate=0.203, sample size=10,537,667)* | | | | | |
| Medicaid  Expansion | -3.695  (1.899) | 0.001  (0.003) | 0.001  (0.001) | 0.001  (0.001) | 0.001  (0.001) |
|  |  |  |  |  |  |
| Full ACA (w/  Medicaid) | -2.273  (1.535) | -0.0003  (0.003) | 0.001  (0.001) | 0.000  (0.000) | 0.001  (0.001) |

Notes: Standard errors, heteroscedasticity-robust and clustered by state, are in parentheses. *** indicates statistically significant at 0.1% level; ** 1% level; * 5% level. Sampling weights are used. All regressions include area and time fixed effects and the full set of controls.

**Table A9. Implied Effects of the ACA at Mean Pre-Treatment Uninsured Rate for the Subsample That Did Not Have a Change in Marital Status in Last 12 Months**

|  | Any insurance | Any private | Employer-sponsored | Individually purchased | Medicaid | Other |
| --- | --- | --- | --- | --- | --- | --- |
| *Non-elderly adults aged 19-64 (pre-treatment uninsured rate=0.203, sample size=10,201,544* | | | | | | |
| Medicaid  Expansion | 0.050***  (0.011) | -0.009  (0.009) | 0.002  (0.007) | -0.010  (0.012) | 0.062***  (0.008) | 0.001  (0.001) |
|  |  |  |  |  |  |  |
| Full ACA (w/  Medicaid) | 0.087***  (0.005) | 0.028***  (0.006) | 0.016***  (0.004) | 0.012***  (0.003) | 0.062***  (0.007) | 0.001  (0.001) |

Notes: Standard errors, heteroscedasticity-robust and clustered by state, are in parentheses. *** indicates statistically significant at 0.1% level; ** 1% level; * 5% level. Sampling weights are used. All regressions include area and time fixed effects and the full set of controls.
